# Workplace violence among Registered Nurses in Saudi Arabia: A qualitative study

**DOI:** 10.1002/nop2.679

**Published:** 2020-11-17

**Authors:** Jordan Tovera Salvador, Friyal Mubarak Alqahtani, Maha Mohammed Al‐Madani, Mu’taman Khalil Jarrar, Sherien Ragab Dorgham, Lilibeth Dela Victoria Reyes, Mohammed Alzaid

**Affiliations:** ^1^ Department of Nursing Education College of Nursing Imam Abdulrahman Bin Faisal University Dammam Saudi Arabia; ^2^ Department of Community Health Nursing College of Nursing Imam Abdulrahman Bin Faisal University Dammam Saudi Arabia; ^3^ Department of Fundamentals of Nursing College of Nursing Imam Abdulrahman Bin Faisal University Dammam Saudi Arabia; ^4^ College of Medicine Imam Abdulrahman Bin Faisal University Dammam Saudi Arabia

**Keywords:** aggression, bullying, Middle East, nurses, qualitative research, Saudi Arabia, workplace violence

## Abstract

**Aim:**

The primary aim of this qualitative inquiry is to explore the experiences of Registered Nurses working in Saudi Arabia, which was a guide to answer the question, “what are the lived experiences of Registered Nurses working in a selected government hospital in Eastern Region of Saudi Arabia towards workplace violence?”

**Background:**

Workplace violence is a social phenomenon that needs a widespread campaign to eradicate. Incidence from all over the world continues to grow in number, especially among Registered Nurses in Saudi Arabia.

**Methods:**

Descriptive phenomenology. Purposive‐convenience sampling was used in selecting 21 Registered Nurses as participants for individual in‐depth interviews. Data were gathered over an 11 month's period from September 2017 to August 2018. We used Colaizzi's method for analysing the data. COREQ criteria were adapted in reporting the results of the study.

**Results:**

Three themes had emerged from the experiences of the participants: *“co‐workers become* unjust and violent”; “socio‐cultural divergence towards healthcare workers”; and “violence from outside influences affecting the workplace.”

## 1 INTRODUCTION

There are several definitions of workplace violence. Based on the US Department of Labor Occupational Safety and Health Administration (2002), workplace violence is defined as:

[…] violence or threat of violence against workers. It can occur at or outside the workplace and can range from threats and verbal abuse to physical assaults and homicide, one of the leading causes of job‐related deaths (p. 1).

Likewise, the Center for Disease Control (2002) defined it as “violent acts” (includes physical assaults and threats) directed towards persons at work or on duty. Despite numerous campaigns and activities, incidences of workplace violence in the world are rampant worldwide including Saudi Arabia (Basfr et al., 2019). This study aims to promote actions to discuss violence in the health sector workplaces particularly in the scope of the nursing profession and complement actions implemented by the different international agencies at different levels in decreasing the incidence of violence in the health sector all over the world.

### 1.1 Background

Studies have shown that episodes of workplace violence had unfavourable effects on both the physiological and psychological aspects of nurses victimized by the perpetrators (Martinez, 2016). These were the reasons why the International Labor Organization, International Council of Nurses, the World Health Organization and the Public Service International (2002) created a framework guideline for addressing workplace violence in the health sector. They had defined workplace violence as

…incidents where staff is abused, threatened or assaulted in circumstances related to their work, including commuting to and from work, involving an explicit or implicit challenge to their safety, well‐being or health (pp. 3).

Numerous workplace violence incidences among healthcare professionals especially among nurses were not reported and studied (Alsaleem et al., 2018; Alshehry et al., 2019; Phillips, 2016). Nurses being the front liners play an essential role in the patient care delivery system, have always been considered as the most vulnerable subject for aggression, bullying and incivility in the healthcare setting in the past decades particularly in the emergency departments, inpatient psychiatric settings and nursing homes (Cheung & Yip, 2017). One evidence that workplace violence was rampant worldwide is the study conducted in Slovenia, out of 692 nurses recruited for a workplace violence survey, the small number of respondents had reported violence in writing form since they believed that in spite of knowing the figure of the victims, nothing will be changed. They would always fear losing their jobs if they tell the truth (Kvas & Seljak, 2014). Another proof that violence in the workplace does happen worldwide was the study conducted among 850 nurses in Hong Kong. A total of 44.6% of respondents revealed that they had experienced various kinds of workplace violence, such as verbal abuse (39.2%), physical assault (22.7%) and sexual harassment (1.1%; Najafi et al., 2018).

In the Middle East, for instance, a study conducted in Palestine revealed 80.4% of workplace violence, of which 20.8% were referred to physical and 59.6% were referred to non‐physical workplace violence among physicians and nurses (Kitaneh & Hamdan, 2012). Furthermore, substantial proportions of nurses have experienced workplace violence in Jordan and Lebanon, with a ratio of 75% and 64.8%, respectively (Alameddine et al., 2015; ALBashtawy & Aljezawi, 2016). These international studies concluded that workplace violence may lead a professional to render poor quality of patient care and this could make huge impacts on the nursing profession. Earlier systematic review study with a total of 3 million participants of the eligible studies found out that 61.9% of healthcare professionals were reported workplace violence (Liu et al., 2019). Despite this fact, various works of literatures and studies were tackling this controversial issue; however, limited information about this phenomenon against healthcare professionals in the Gulf Cooperation Council (GCC) countries is available.

In Saudi Arabia, in line with Vision 2030 and the Ministry of Health's (MOH) endeavour to provide and raise the level of health services for all citizens, many achievements have been made, including an increase in healthcare facilities to provide comprehensive and accessible healthcare services (MOH, 2018a). Despite these efforts and achievements, programmes to mitigate workplace violence among healthcare professionals are needed. Nurses are most of the healthcare workforce in Saudi Arabia, with a total of 82,505 nurses in the public sector and 44,708 in the private sector (MOH, 2018b). Violence against nurses had been a major challenge for the hospital directors particularly with its aftermath negative impacts resulting in poor performances and low self‐confidence and esteem (Alkorashy & Al Moalad, 2016). Based on the MOH annual report 1437‐1438H (2016‐2017G), expatriate nurses represent 40% (with a total of 32,832) of the nursing workforce in the public sector and 94.5% (with a total of 42,258) in private sector in Saudi Arabia (MOH, 2018b, pp. 76 & 376). Expatriate nurses refer to all non‐Saudi nurses. A study reported by Alhusain et al. (2020) revealed that non‐Arabic speaking healthcare workers are more likely to be exposed to violence than Arabic speakers. In a study conducted among 738 healthcare workers in 2 government health facility in Abha City, Saudi Arabia, 57.5% of the respondents were victims. Verbal assaults and slaps were the most common forms of violence (Al Anazi et al., 2020; Alsaleem et al., 2018). Another study conducted in a Saudi university hospital found out that almost half of the total sample quota of 360 nursing personnel had experienced violence during the 12‐month period before the study was formally conducted using the Massachusetts Survey on Workplace Violence/Abuse (Alkorashy & AL Moalad, 2016). Perpetrators were identified based on countless observations and research investigations. Surprisingly, almost the same worldwide such as patients (36.6%), relatives (17.5%), co‐workers (7.7%) and supervisors (6.3%; Cheung & Yip, 2017; Najafi et al., 2018). Common reasons why perpetrators lead to violence were due to understaffing, misunderstandings, long cue of services, lack of staff training and absence of policies of workplace violence (Alkorashy & AL Moalad, 2016). As mentioned, only limited existing literatures tackle much of the experiences and feelings of workplace violence victims, which is why this study leads one step ahead from exploring ways and means to eradicate this controversial social phenomenon in the health sector.

The significance of workplace violence among nurses was more than the priority for policymakers, researchers and nurse managers locally and internationally. Most studies investigated workplace violence were conducted in Asia, Europe and North America. Limited information about workplace violence against healthcare professionals in the Gulf countries. Interestingly, most of the published studies in the Middle East and Saudi Arabia were reported the forms of workplace violence. For instance, studies conducted in Jordan, Lebanon and Saudi Arabia found the verbal abuse is the most vulnerable workplace violence (ALBashtawy & Aljezawi, 2016; Alqahtani et al., 2020). However, limited studies investigated the perpetrators of workplace violence in the Middle East. In Oman, visitors and patient's family members were the most raiders to workplace violence (Al‐Maskari et al., 2020). This study reported here is the first qualitative study reported narratives of Registered Nurses who had first‐hand experiences of the perpetrators of workplace violence in Saudi Arabia. Enumerable significance can prop‐up to various areas of nursing: (a) it undermines retention and recruitment initiatives, which are the most commons predicaments in the nursing practice; (b) it initiates discovery of new strategies (e.g. innovative reporting scheme, mobile application) on how to fully eliminate workplace violence in the health sector through continuous nursing research; (c) it provides early training for students either theoretical or clinical simulations, thus, incorporating workplace violence in the nursing education curriculum; (d) it allows the participants to voice‐out their lived experiences that could prevent other Registered Nurses to experience the same; and (e) it spearheads nurse practitioners, managers and policymakers in Saudi Arabia to come up with specific, measurable and realistic interventions to eradicate this growing social phenomenon, especially in the health care.

Exploring the experiences of Registered Nurses working in Saudi Arabia is the primary goal of this qualitative investigation. This research was guided to answer the question: “what were the experiences of workplace violence of Registered Nurses working in Saudi Arabia?”. Understanding the workplace violence experiences of nurses would encourage professional organizations and educational facilities to prepare nurses against workplace violence and suitable interventions would be implemented to minimize the incidence of the violent acts of this social phenomenon (Kvas & Seljak, 2014).

## 2 MATERIALS AND METHOD

### 2.1 Research design

In this paper, descriptive phenomenology was chosen because the lived experience is understood from a lifeworld approach coming from the writing of Husserl as how individuals give meaning to experiences towards a certain phenomenon and its applicability in understanding the voices of the participants (Creswell, 2013; Dahlberg et al., 2008; Salvador, 2016a). Understanding lived experiences is based from the lifeworld, which forms the ontological and epistemological foundations of the study that is underpinned from the participant's awareness of the lifeworld, their bodily being in the world and how they interact with others (Sundler, Lindberg, Nilsson, & Palmer, 2019). Besides, the entire study was guided by the methodological principles of questioning pre‐understanding, adopting a reflective attitude and emphasizing openness. In analysing the data, thematic analysis was used to come up with better understanding of patterns of meaning from the gathered descriptions of the experiences.

### 2.2 Participants and sampling

Purposive‐convenience sampling was used in selecting the 21 participants (11 males and 10 females). Purposive sampling (judgment) is the intended option of a participant due to the standards the participant have, which also involves identification and selection of a person or groups that are well‐informed towards a phenomenon of interest (Creswell, 2013; Etikan et al., 2016), while convenience is a class of non‐probability sampling that does not create statistical data, but aims to allow the phenomenon to occur. This was to find shared or mutual dimensions that cut through a varied sample while at the same time propose the prospect to detail unique or diverse variations (Bricki & Green, 2007; Patton, 2002). Participation in the study was purely voluntary and no conflict of interest between the researchers and the participants (Salvador and et al., (2020).

The demographic profiling of the enrolled participants can be seen in Table 1. The Registered Nurses were currently working in a government hospital in the Eastern Region of Saudi Arabia. Casual talks between the researchers and participants were done to establish rapport (Salvador, 2016b). The researchers explicated the purpose and flow of the interviews before signing the consent. The inclusion criteria included the following: (a) a Registered Nurse with a minimum of 2 years’ experience as a Registered Nurse; (b) had an experience of violence in any form; (c) willingness to impart personal experiences; and (d) able to speak the English language. Each participant was expected to share personal experiences regarding the phenomenon under investigation that would provide new insights to the readers (Salvador & Alqahtani, 2019). Lastly, the researchers had chosen 2 years as work experience in the hospital because at this time, Registered Nurses had already adapted to the hospital environment and surpassed the probationary period of 6 months. This would enable them to see a comparison between the first year to the succeeding years as Registered Nurses towards workplace violence.

**Table 1:** Demographic profile of the participants

No.	Pseudonym	Nursing department	Age	Gender	Country of origin	Years in nursing practice	Civil status
1	Blue	Emergency	29	M	Philippines	5	Single
2	Yellow	Outpatient	43	F	South Africa	20	Married
3	Pink	Delivery	37	F	Egypt	14	Married
4	Red	Operating	28	M	Saudi Arabia	3	Single
5	Magenta	Ob‐Gyne	32	F	India	8	Single
6	Indigo	Intensive care	30	M	Egypt	6	Married
7	Lilac	Paediatric	31	F	Saudi Arabia	7	Married
8	Brown	Surgical	42	M	Philippines	21	Married
9	Pearl	Paediatric	34	F	Saudi Arabia	7	Married
10	Old Rose	Emergency	46	F	South Africa	23	Married
11	Tan	Surgical	23	M	Saudi Arabia	2	Married
12	Black	Haemodialysis	37	M	Philippines	15	Married
13	Orange	Ob‐Gyne	39	F	Egypt	16	Married
14	Bronze	Emergency	34	M	India	9	Married
15	Jade	Newborn	27	M	India	3	Single
16	Violet	Intensive care	28	F	India	4	Single
17	Gold	Medical	44	F	South Africa	21	Married
18	Sage	Surgical	38	M	Egypt	15	Married
19	Silver	Haemodialysis	30	M	Philippines	6	Single
20	Fuchsia	Newborn	33	F	India	8	Single
21	Grey	Psychiatric	53	M	Philippines	30	Single

### 2.3 Interviews

Data were gathered over 11 months from September 2017 to August 2018 through individual interviews. Individual in‐depth phenomenological interview was used to probe the participant's attitude, beliefs, desires and experiences to provide better comprehension of the phenomenon. Initially, the research team piloted a “subject‐object interview” to 3 Registered Nurses in the selected government university hospital who met the inclusion criteria and used as a basis in creating an interview protocol that includes questions and probes to use for follow‐up seen in Table 2 (Bryman, 2012; Dikko, 2016). The Registered Nurses enrolled in the pilot interviews were excluded from the actual study. Before conducting individual interviews, all researchers underwent workshops about proper approach and techniques in conducting the interview sessions, which includes peer debriefing, transferability, triangulation of data and various techniques in handling data and analysis. After 2 months, the researchers revisited and directed individual interviews (Bauger & Bongaardt, 2016). The schedule of interview depends on the availability and most suitable time of the participants, usually during off‐duty hours and/or before and after scheduled shifts. Interview sessions were conducted 30–45 min and were conducted in various places depending on the preferred places of the participants where they can feel comfortable and safe in sharing their lived experiences about workplace violence (e.g. coffee shops, restaurants and empty and secluded hospital waiting areas; Salvador et al., 2016).

**Table 2:** Semi‐structured interview guide

Interview questions	
Can you please tell me something about yourself? How would you describe yourself as a nurse and co‐worker? Can you please tell me about your idea/s about workplace violence? Can please you tell me some instances that you experience workplace violence. How would you describe your social interaction with your co‐workers, patients and patients’ significant others? How do you cope and handle stress and anxiety in the workplace?	How does your institution handle conflict resolution? Does your institution have workplace violence watch or patrol? How does your immediate supervisor handle such case and how it was dealt? How would you describe yourself as a nurse, as a person with human rights, and as a person with dignity? How does this experience change your personal and professional life/endeavour? What are your plans and expectations for the future? What are your final thoughts regarding your experience?

### 2.4 Data analysis

The data saturation process was patterned according to Colaizzi's seven steps in analysing qualitative data. Initially, the researchers started analysing by reading and re‐reading individually the transcript to draw the general sense of the conversation. The significant statements identified in relation to the phenomenon were extricated, which was followed by a series of discussions of the meanings formed from the statements. To eliminate possible biases, each researcher's pre‐conceived ideas were placed in brackets, to prevent a theoretical‐deductive approach and focus on an inductive process (Salvador et al., 2019). The word files and excel spreadsheets containing the researchers were shared and arrived in the final synthesis; thus, themes generated were identified based solely on the data analysis. The formulated meanings were clustered until such time it cannot anymore be categorized. The final deliberation of the results came up with 3 major themes. In cases of disagreement, the researchers go back and discuss the data analysis and final results will be based on the consensus of the research team usually done by casting votes. The final outcomes were immersed into an “*exhaustive description”* to fully uncover the fundamental structure of the phenomenon. Lastly, results were discussed among the participants for member checking (Colaizzi, 1978).

### 2.5 Rigour

To guarantee the rigourously scientific nature of this qualitative investigation, 4 concepts were thoroughly considered: credibility, confirmability, dependability and transferability (Salvador et al., 2019). To ensure credibility, researchers transcribed the interviews on the same day to prohibit any form of vagueness and ambiguity, all results were validated from the transcript files, field notes and audio‐recorded individual interviews. Reflective journals were used to recognize any personal biases. Moreover, the researchers also sought three hospital staff nurses who were not enrolled in the study to validate the emerging essence of the phenomenon compared with their own experiences through a referral system from nursing colleagues from different countries. The following are the criteria of selection: (a) a Registered Nurse who experienced workplace violence first‐hand; (b) willingness to share insights about the results of the study to personal experience; and (c) can speak the English language. These Registered Nurses were from the Philippines, the USA and Saudi Arabia. This process enabled the researchers to confirm that findings can happen not only in one place, which would attest to the study's transferability. For dependability, the researchers ensured that all processes throughout the study were recorded for consistency and reliability, so whenever an individual checks it, similar results will be generated (Polit & Beck, 2012). Three independent peer reviewers were invited to double checked the relevancy, accuracy and meaning of the data for conformability. Peer reviewers have expansive experiences in nursing education, practice, administration and research. Specific criteria were as follows: (a) 10 years in the nursing profession; and (b) with expertise on qualitative research with multiple number of publications. All reviewers have similar perspectives towards the objectivity of the study. More so, participants were also given the chance to check on the theme clusters and major themes (fittingness) by using direct quotations to embody how the phenomenon was perceived (Lincoln & Guba, 1985; Polit & Beck, 2012).

Finally, in reporting the results, Consolidated Criteria for Reporting Qualitative Research (COREQ) criteria (Tong et al., 2007) was adapted. All interviews were audiotaped, transcribed into verbatim on the same day and kept in a case for proper safekeeping and future reference. Participants were well‐informed of the confidentiality and data will be destroyed and disposed securely once it is no longer needed, after agreed periods of retention have expired per the University's Information Handling Policy.

### 2.6 Ethical considerations

This study was approved by the Institutional Review Board of Imam Abdulrahman Bin Faisal University (IAU 2017‐134). The researchers also sought permission to conduct a study in the selected government university hospital. All requirements were submitted (IRB certification, a brief summary of the study, informed consent and interview questions). After several weeks, approval to conduct the study was given to the research team. A memorandum was given to the nursing service office to circulate in all nursing departments that interviews will be conducted. Prior to data gathering, participants signed informed consent that included permission to publish quotes. Participants were given the autonomy to express and verbalize their lived experiences as well as other concerns (e.g. emotional, moral, etc.). Individuals who were a priori vulnerable were not interviewed (e.g. mental and psychological problems). Participants who were unintentionally become a posteriori vulnerable were given counselling from the hospital's counselling department during or after the duration of the interviews (Larsen et al. (2008).

## 3 RESULTS

After extensive meetings and deliberations with the selected panel of reviewers, the researchers came up with 3 themes and 9 theme clusters that reflected the workplace violence experiences of the participants given in Table 3.

**Table 3:** Theme clusters and emergent themes

Theme cluster	Themes
Assaulting junior staff nurses by senior staff nurses	Co‐workers become unjust and violent
Stereotyping ageing workforce	
Experiencing peer‐to‐peer violence	
Public humiliation due to miscommunication	Socio‐cultural divergence towards healthcare workers
Racial discrimination among expatriate workers	
Wrong impression of men in nursing	
Threatening co‐workers by a loved one	Violence from outside influences affecting the workplace
Cyberbullying	
Physical, verbal attacking and mobbing	

### 3.1 Co‐workers become unjust and violent

This major theme depicts the workplace violence experiences of the participants with their co‐workers. In their accounts, the perpetrators do not only pertain to the co‐nurses, but also from various healthcare professionals and departments. Three theme clusters emerged:

(1) “assaulting junior staff nurses by senior staff nurses” refers to verbally assaulting junior nurses typically in their probationary periods usually 6 months on their poor performances, lack of initiative, incompetence, bad attitude and insubordination:

I am always monitored and scolded by my senior nurses for all the things I do in the unit. Sometimes. I am afraid of the expectations from me as a newly staff nurse. It comes to a point that I feel weak and stupid because of the harsh comments I hear from them…. (P7)

(2) “stereotyping ageing workforce” involves creating an environment with the stigma that older generation cannot contribute to the success of the group and treated as liabilities and burden, since they had limitations in their physical, skills and cognitive aspects:

As part of the senior staff nurses in terms of age. I feel somehow left out and sometimes single out since I was different opinions and perceptions compared to the young ones. What we shared and have in the past is really different from what we have today. The value of respect to older staff nurses is gone. I have no choice but to accept it…. (P21)

and (3) “experiencing peer‐to‐peer violence” pertains violence coming from different employees in the hospital such as doctors, medical technicians, administrators, etc.:

Some of the doctors are not so cooperative with us. Most of them think that we are just nurses. We feel that we should be respected as we respect them. Honestly, it is not only the doctors, other departments have their own share of not so good experience. I just hope, we will be able to mend these differences…. (P14)

### 3.2 Socio‐cultural divergence towards healthcare workers

This major theme illustrates the workplace violence experiences with their respective patients, client's family members and visitors. Three theme clusters were generated:

(1) “public humiliation due to miscommunication” refers to language barriers, body languages and cultural gestures causes some people to misunderstood what nurses want to say resulting in verbal clashes, commotions and worst physical attacking:

During my first 3 years in the hospital, it was really hard for me to adapt the situation because I cannot speak and understand the Arabic language. There was an instance that my patient got upset because he cannot understand me and told me ‘mafi muk’ (Arabic). I asked my co‐worker about the meaning of that phrase, to my dismay I found out that the meaning of it was ‘no brain’. I felt humiliated and insulted but what can I do. I just kept silent and did not mind it happened. Somehow, my self‐confidence went down. I am questioning myself, if my decision of coming here is right or wrong…. (P12)

(2) “typecasting nurses due to religious disparity” embraces religious practices and customs that serve as guiding principles for people, which affected on their interaction with nurses with dissimilar religion:

It is known for a fact that Saudi Arabia is known to have rich culture and traditions. However, in the healthcare delivery system, somehow, people should be accepting. There are some instances that because of no familiarization with their cultural, social and religious practices, they will be telling you ‘not so good things’. Whenever they utter things like that, I feel ashamed of myself…. (P2)

and (3) “wrong impression of men in nursing” pertains to the socio‐cultural ideology that the nursing profession is only for females:

There was a time I am in‐charged of a local patient. The patient was not cooperative because I am literally an expat. His family was not happy too, they raised their voices and tell me that they wanted a female nurse or someone who could understand them unlike me who has different backgrounds…. (P8)

### 3.3 Violence from outside influences affecting the workplace

This major theme portrays the workplace violence experiences that involve some personal and professional involvements with loved ones, family members, friends and acquaintances whom the participants had various close encounters happening inside and outside the hospital premises. Three theme clusters emerged from this theme:

(1) “threatening co‐workers by a loved one” refers to personal issues from family and friends, which resulted in harassment, mocking, abuse and/or any kind of violence happening in the workplace causing someone to feel uncomfortable and uneasy:

My husband got angry because of rumors and gossips bout my involvement with one male staff nurse who happens to be her ex‐boyfriend. In fact, he was just a friend. They started spreading bad news about me and single me out during duty. And someone informed my husband. My husband confronted the involved person and almost had a fight…. (P13)

(2) “cyberbullying” pertains to an intentional social media attacking that may lead to character assassination and public scandal:

Some of the staff nurses expressed their rants and emotions thru social media especially in Facebook. Although most of the time, no names were mentioned but people from the hospital or unit would easily notice who the person is referring to. Gossips and hearsays are usual occurrence after posting in the social media. I was victimized twice, at first I ignored it but the last one, I confronted the person but that person kept denying it…. (P9)

(3) “physical, verbal attacking and mobbing” refers to violence experiences of nurses from an individual or group either by harming them intentionally or unintentionally:

When I was heading outside the gate of the hospital towards my home, a man suddenly attacked me without any reason at all. I was shouting for help. No one was there. It is really scary and dangerous. Aside from that they got my bag that contained my essential personal belongings, good thing I had my purse on my back pocket…. (P19)

There were group of teenagers who mobbed me when I am about to go to my flat. I was really scared and tried my best to run but they were throwing stones that caused me some bruises. (P14)

## 4 DISCUSSION

The lived experiences shared by the participants in this qualitative investigation are quite similar with the types of workplace violence described by the National Institute for Occupational Safety and Health (2016) such as workplace violence from worker‐on‐worker, criminal intent, customer/client and personal relationship. Likewise, these may come in various forms in the hospital such as verbal (ranging from offensive commentaries, inappropriate jokes, etc.), physical (assaults and battery), gesture (non‐verbal threatening and body languages like eye‐rolling, glaring, etc.), exclusion (intentional isolation) and cyber (multimedia mocking, threatening, etc.)

Workplace violence between co‐workers is a worldwide phenomenon and common in every healthcare facility. This could be in various forms of aggression and bullying that often manifests as verbal abuse and emotional torture, which is cruel, rude, vindictive, humiliating and/or offensive (NIOSH, 2016). Nurses tend to be violent for innumerable unexplainable reasons. This is mostly coming from fellow staff nurses rather than from their immediate supervisors (Granstra, 2015). This kind of workplace violence in the nursing profession is called “horizontal bullying or lateral violence” and currently investigated as one of the most alarming issues in the healthcare profession today. This type of violence is viewed as being “the lower on the food chain” such as in a manager to staff or physician to staff nurse though the incidence of peer‐to‐peer violence is also common as shared by the participants (NIOSH, 2016). It is not only in the hospital that this phenomenon can be experienced, but it can also happen in any professional setting like academic institutions, primary health clinics, private institutions. Unfortunately, not all hospital personnel recognize it especially in special areas like the operating rooms and emergency departments (Chipps et al., 2013). Since the operating room environment is competitive and “*toxic,*” healthcare workers viewed misbehaviour and incongruous attitude from workers as normal. One participant emphasized that awareness among healthcare professionals should be imposed to let them know the nature of this phenomenon to prevent it from occurring.

Another common lived experience of violence shared by the participants and considered as the most common in a healthcare setting is the “socio‐cultural divergence towards healthcare workers” especially in Saudi Arabia where most of the workforce in the hospitals are expatriates. Comparable to the participants’ experiences, a study conducted in India mentioned that the client and their family members become aggressive and violent to nurses and junior residents who have less clinical experiences, comparatively less age group and male gender, which resulted in physiological and psychological damages on the healthcare provider (Sachdeva et al., 2019). Similarly, 70.6% and 43.1% of workplace violence incidents in a public hospital in Iran were from patients and companions respectively. Unrealistic expectations were the most common attributing factors leading to verbal abuse, threats, physical abuse, sexual abuse and ethical discriminations (Honarva et al., 2019). The study concluded detrimental effects not only for healthcare providers but as well as university lecturers causing them to lose their self‐confidence, composure and competency bringing serious repercussions in their work areas (Gupta et al., 2017). Thus, this shows a worldwide phenomenon that usually happens in any hospital department commonly in psychiatric and emergency care facilities, hold backrooms and geriatric facilities; however, it is not limited to these areas.

“Violence from outside influence affecting workplace” was identified as the last kind of lived experience identified by the participants. The perpetrator of violence can also come from people within the nurse's circle of friends, relatives, acquaintances and/or anyone not related to the victim. This type of violence can be a source of physical and psychological violence at work coming from family problems, relationship with other people, unsettled transaction and businesses; thus, its major effects may lead to deprived physical and physiological health, amplified psychological stress and decreased employment fulfilment (Brewer & Whiteside, 2012). Moreover, the perpetrator may also have no legal connection to any of the workplace employees and is intently attributed to crimes such as kidnapping, robbery, trespassing and even mobbing resulting in physical violence. Campo and Klijn (2018) mentioned that there was a low percentage of reported violence since healthcare professionals believed that this would be useless, insignificant and will not be given appropriate action by the administrators.

CDC has enumerated common causes of workplace violence with the following reasons, which were consequently evident in the participants’ experiences: (a) higher patient morbidities, encounter to violent persons, shortages in the staff and the absence of robust prevention activities and safekeeping protocols are all hindrances in decreasing violence against healthcare professionals; (b) any form of violence can happen anywhere inside and outside the hospital, however, the incidence are frequently in emergency and psychiatric units, waiting areas and an adult care facility; and (c) the location and vicinity of the healthcare setting contribute to the safety of the employees. Despite numerous awareness campaigns to successfully eliminate it, more and more cases were being reported either direct (verbal and physical) or indirect (relational and psychological) ways due to unsustainable implementations (Karatuna, 2015). Anti‐workplace violence programmes were reported to decrease the incidence among nurses in the hospital at one period, however, 6 months after the implementation, it started to increase once again due to lack of leadership and management and proper way of handling cases of violence still needs to be improved (NIOSH, 2016; Stagg et al., 2013).

### 4.1 Limitations

The study solely investigated the experiences of nurses and does not involve any of the perspectives of the nursing administrators or the behaviour of the perpetrators.

## 5 CONCLUSIONS

Workplace violence is a global phenomenon that leaves detrimental and unfavourable effects to both private and public healthcare professionals, particularly among Registered Nurses who are considered as the front liners in any healthcare setting. Saudi Arabia has the same issues just like any other country facing this kind of social problem, which nursing managers and advocates need to act on for eradication or if not at least a continuous decrease in number each year. Three themes had emerged from the experiences of the participants: “co‐workers become unjust and violent; socio‐cultural divergence towards healthcare workers; and violence from outside influences affecting workplace.” Knowing the narratives of Registered Nurses who first‐hand experience workplace violence would give awareness to nurse managers to come up with specific, measurable and realistic interventions to eradicate this growing social phenomenon especially in health care. Therefore, the study would like to conclude that the lived workplace violence experiences of Registered Nurses working in Saudi Arabia increases an individual's understanding of the subject, which may contribute to finding collective resolutions to eliminate this social phenomenon. Nurses, as well as other healthcare providers, must need to feel safe in the workplace to fully function as a healthcare provider and to deliver the best quality service for the clients (Bent, 2016).

### 5.1 Recommendations

Establishing a robust and systematic multi‐streaming initiative should be implemented to safeguard the nurses from any possibility of harm in the clinical settings that include setting up a powerful violence prevention and training programme for which employees are well‐oriented such as establishing policies for its occurrence and near misses reporting, recording and screening that may encourage an atmosphere of confidence and respect between staff and management. Creating a comprehensive physical and psychological counselling and debriefing plan for employees who encountered any form of abuse or aggression‐related issues and the provision of trauma‐informed treatment. For nurse managers, creating workplace surveys may help recognize various types of issues encountered by employees in their daily jobs. It would also be helpful to design an assessment tool for employment hazard analysis that focuses on job tasks to spot hazards associated with workplace aggression, incivility and violence and/or maybe modified to cut back the likelihood of violence occurring.

Patients can also engage in the detection of reasons for violence, day‐to‐day events contributing to violence and successful response. Remodeling of workplace environment, such as re‐arranging the work areas and stations that would eliminate hazards at work or build barriers between the worker and the hazards as well; door locks are used to minimize risk exposure for employees; better or more lighting and more open exits. Finally, developing training programmes for new nurses before assigning jobs. All nurses should also be trained regularly to improve their health awareness and safety and prepare them on how to respond to the warning signs of workplace violence.

## CONFLICT OF INTEREST

The authors have no conflict of interest to declare.

## AUTHOR CONTRIBUTIONS

All authors conceptualized and analysed the data and corrected the final draft of the article. Likewise, all authors helped together in collecting the data as well as in the analysis and interpretation. Final draft of the article was reviewed and approved by all authors in a roundtable discussion. Ithenticate was utilized in checking the similarity index.

## ETHICAL APPROVAL

This study was approved by the Institutional Review Board of Imam Abdulrahman Bin Faisal University (IRB approval number: IAU 2017‐134). Prior to data gathering procedures, informed consents were collected and signed by the participants. The informed consent included permission to publish quotes. Moreover, autonomy was highly‐observed throughout the process as well as participants’ confidentiality. The researchers made sure that participants has the freedom to withdraw whenever they feel uncomfortable and unsafe. Participation was purely voluntary and no conflict of interest between the researchers and the participants.

## Data Availability

The date sets used for the current study are available from the corresponding author on reasonable request.
